# Human Endogenous Retroviruses (HERVs) and Mammalian Apparent LTRs Retrotransposons (MaLRs) Are Dynamically Modulated in Different Stages of Immunity

**DOI:** 10.3390/biology10050405

**Published:** 2021-05-05

**Authors:** Maria Paola Pisano, Nicole Grandi, Enzo Tramontano

**Affiliations:** Laboratory of Molecular Virology, Department of Life and Environmental Sciences, University of Cagliari, 09042 Cagliari, Italy; mp.pisano@unica.it (M.P.P.); nicole.grandi@unica.it (N.G.)

**Keywords:** HERV, MaLR, immunity, RNA-seq, vaccine, differential expression

## Abstract

**Simple Summary:**

Human Endogenous retroviruses (HERVs) and Mammalian Apparent LTRs Retrotransposons (MaLRs) are remnants of ancient retroviral infections that make up about 8% of the human genome. HERV and MaLR expression is regulated in immunity, and, in particular, is known for their up-regulation after innate immune activation. In this work, we analyzed the differential expression of HERVs and MaLRs in different stages of immunity, triggered by the administration of an inactivated vaccine.

**Abstract:**

Human Endogenous retroviruses (HERVs) and Mammalian Apparent LTRs Retrotransposons (MaLRs) are remnants of ancient retroviral infections that represent a large fraction of our genome. The HERV and MaLR transcriptional activity is regulated in developmental stages, adult tissues, and pathological conditions. In this work, we used a bioinformatics approach based on RNA-sequencing (RNA-seq) to study the expression and modulation of HERVs and MaLR in a scenario of activation of the immune response. We analyzed transcriptome data from subjects before and after the administration of an inactivated vaccine against the Hantaan orthohantavirus, the causative agent of Korean hemorrhagic fever, to investigate the HERV and MaLR expression and differential expression in response to the administration of the vaccine. Specifically, we described the HERV transcriptome in PBMCs and identified HERV and MaLR loci differentially expressed after the 2nd, 3rd, and 4th inactivated vaccine administrations. We found that the expression of 545 HERV and MaLR elements increased in response to the vaccine and that the activation of several individual HERV and MaLR loci is specific for each vaccine administration and correlated to different genes and immune-related pathways.

## 1. Introduction

A large proportion of our genome derives from retroviral infections that took place millions of years ago [1]. The proviral genome of these retroviruses has been integrated within the DNA of germline cells, being transmitted to the offspring and then fixed in the human population during primate evolution [2]. These LTR-retrotransposons now make up about 8% of the human genome, including Human Endogenous Retroviruses (HERVs) and Mammalian Apparent LTRs Retrotransposons (MaLRs) [3]. At the time of integration, the HERV genome was composed of four retroviral genes (*gag*, *pro*, *pol*, and *env*) flanked by two LTRs, while the MaLRs genome was similar but lacked the *env* gene [3,4]. LTR-retrotransposons accumulated several mutations over time, and solitary LTRs were generated by recombination occurrences [3,4,5]. Despite that, both solo LTRs and proviral MaLRs/HERVs can contribute to human biology and development [6,7,8]. Indeed, the sequence of solo- and proviral LTRs includes enhancers, promoters, and polyadenylation signals that may modify the expression of neighboring cellular genes [6,9,10]. Moreover, some HERV loci maintain intact retroviral ORFs and are expressed in human tissues [6,11,12]. The HERV transcriptional and translational activity has been hence investigated, especially for its possible involvement in pathogenesis [13]. In particular, several studies have proposed the role of HERVs in cancer and autoimmunity [13,14,15,16,17,18]. In general, the HERV expression is increased in tumor cells [15,19] and HERV Env might act as fusogens and be potentially linked to tumor development [20]. Moreover, two accessory proteins (namely Np9 and Rec) that are coded from doubly spliced *env* transcripts are proposed to have oncogenic properties [13,21,22,23]. Besides tumorigenesis, an Env protein is potentially associated with multiple sclerosis, as it has been shown to induce inflammatory effects and hold superantigen activity [13]. By consequence, a monoclonal antibody recognizing this protein is under clinical trial as a possible treatment for multiple sclerosis [24]. However, despite all these studies in the field, no clear causal-effect associations between HERVs/MaLRs and any human diseases are available up to date.

A connection between HERVs/MaLRs expression and the immune response has also been reported [25,26]. The cellular Pattern Recognition Receptors (PRRs) can recognize HERV and MaLR products as Pathogen Associated Molecular Patterns (PAMPs), inducing the activation of the innate immune response [25,26]. Of note, it has been hypothesized that these interactions with PRRs have positively contributed to shaping the evolution of the immune response [27,28]. Moreover, several HERV and MaLR loci are modulated in some inflammatory settings [29,30,31]. Inflammatory-specific HERV/MaLR transcriptional response has been suggested following severe infection, trauma, and burn [30]. Accordingly, the activation of the innate immunity through LPS, TNF-α, and Interferon-γ (IFN-γ) can increase the HERV and MaLR expression, as observed in in vitro and in vivo models [30,31]. The HERV/MaLR modulation after LPS stimulation is similar and correlated to one of the colocalized immune-related cellular genes. This co-localization is interesting for possible interactions between LTR-retrotransposons and the immune response, introducing a possible role of HERVs/MaLRs in regulating the expression of immunity-related genes [29,30,31], and also suggests possible applications of HERVs and MaLRs as biomarkers in immunity settings [31].

Many questions on the role of HERVs and MaLRs in immunity are—however—still unsolved. For example, while it is known that there are several HERV and MaLR up-regulated in response to the activation of innate immunity, it is not clear if the same loci are also expressed and up-regulated in further stages of immunity. We previously reported the activation of specific HERV and MaLR loci after in vivo stimulation with LPS, showing a general HERVs and MaLRs up-regulation in the first stages of the innate immune response. Hence, to analyze the expression of LTR-retrotransposons during the following development of adaptive immune response, we studied the HERV and MaLR transcriptome in PBMCs from individuals being administered with inactivated Hantaan virus vaccine (Hantavax™ [32,33]), vaccination against the Hantaan orthohantavirus (HTNV), the causative agent of Korean hemorrhagic fever. This dataset was publicly available and provided the effects of the administration of an inactivated virus at different time points, following 0–1–2–13 months vaccination schedule. Hence, we used an RNA-seq pipeline to assess HERV and MaLR expression and modulation after each of the three consecutive vaccine administrations, trying to analyze the patterns of HERV and MaLR activation in the various steps of adaptive immunity.

## 2. Materials and Methods

Analyses were based on an RNA-seq public dataset available in the Gene Expression Omnibus (GEO) repository with the accession ID GSE120115. It includes the transcriptome of PBMCs from healthy individuals (*n* = 19) 1 day before the 1st and 72 h after the 2nd, 3rd, and the 4th administration (following a 0–1–2–13 months vaccination schedule) of Hantavax^TM^ inactivated vaccine against Hantaan virus.

HISAT2 Version 2.1.0 (http://daehwankimlab.github.io/hisat2/download/ accessed on 4 February 2021) [34] with default parameters was used to map reads to the reference genome assembly hg38, and the quality of the alignments was evaluated by the stats function of bamtools 2.0.1 [35]. The reads mapping to HERVs/MaLRs and genes positions were quantified through the “union” mode in htseq-count [36], using as reference the coordinates included in the hervgdb4 database [37], the HERV coordinates from Vargiu et al. [3], and gencode.v27 [38] for HERVs/MaLRs, best-preserved HERV proviruses, and cellular gene annotations, respectively. The raw read counts obtained for hervgdb4 fragments, best-preserved HERV proviruses, and genes were then used to quantify their expression level as Transcripts Per Million (TPM), which takes into account both sequencing depth and coding sequence length. We selected the hervgdb4 fragments and HERV proviruses with at least 1 read in at least 26 out of 76 samples. We used a not restrictive threshold to be sure not to exclude from the basal HERV/MaLR transcriptome the elements low expressed in some samples. We performed the differential expression analysis on the kept hervgdb4 fragments and proviruses.

Rlog normalization on human genes and HERV/MaLR raw counts were performed with the DESeq2.v.1.18.1 R (version 3.4.4) package [39]. Normalized counts were used to assess the interpersonal variability through Principal Components Analysis (PCA) and Heatmap generation. The PCAs were built with the function plotPCA in DESeq2.v.1.18.1 and visualized using ggplot2 3.0.0 in R (version 3.4.4). The Heatmaps were built using the top 1500 rlog counts of hervgdb4 fragments with the higher standard deviation across samples, through the pheatmap 1.0.10 R package (version 3.4.4), and considering the correlation distance across samples in a column.

The differential expression analysis of cellular genes, HERVs, and MaLRs was done using DESeq2.v.1.18.1 R package (version 3.4.4) [39] on the raw counts, applying a statistical threshold (False Discovery Rate (FDR) ≤ 0.01 and absolute values of log2 Fold Change ≥ 1) to identify the modulated elements. Plots were built with ggplot 3.0.0 in R (version 3.4.4); while Gene Set Enrichment Analysis (GSEA) was performed with the software fgsea (version 3.12) [40]. Normalized enrichment score (NES) and false discovery rate (FDR < 0.05) were used to quantify enrichment magnitude of cellular genes to the Hallmark immunity pathway database [41], selecting the hervgdb4 fragments correlated (correlation coefficient ≥ 0.9) to the genes enriching these pathways.

## 3. Results

### 3.1. Description of the HERV and MaLR Transcriptome in PBMCs

We used an RNA-seq pipeline to analyze the public RNA-seq dataset GEO: GSE120115, including transcriptomic data from 19 subjects vaccinated against the Hantaan virus, which is causative of hemorrhagic fever with renal syndrome. The vaccine (Hantavax™), is based on an inactivated virus, and the subjects have been vaccinated with four administrations, according to the 0–1–2–13 month schedule [42]. The samples were collected one day before the 1st and two days after the 2nd, 3rd, and 4th vaccination [42], for a totality of 76 samples (Figure S1). Based on neutralizing antibody titers, subjects were classified into non-responders, low responders, and high-responders [42]. We analyzed this dataset, to investigate the HERV and MaLR expression and differential expression in response to the administration of the vaccine, using the hervgdb4 database that includes 197,341 HERV and 227,174 MaLR loci [37]. Within this database, created for the design of Affymetrix HERV-V3 array probes, the HERV and MaLR proviruses and solo LTRs were included as fragments (single genes or functional portions of LTRs), here named hervgdb4 fragments [10]. Moreover, we could discriminate 3167 proviral sequences, which are the best-preserved HERV loci in the human genome assembly, as detected by the software RetroTector [43] and then collected, classified, and characterized by Vargiu et al. [3].

**Figure 1:**
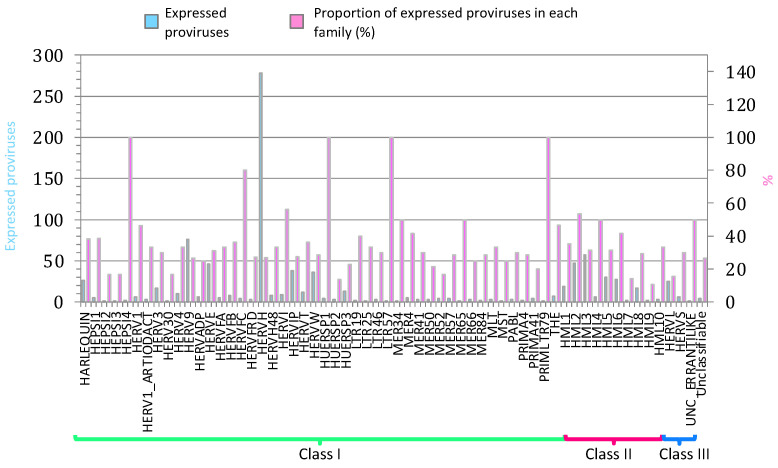
ReTe HERV transcriptome in PBMCs. Basal expression of the most intact HERV loci as reported in Vargiu et al. All the expressed elements are grouped by retroviral classes and groups. Light blue bars indicate the absolute number of expressed proviruses in each retroviral group, while pink bars indicate the percentage of expressed proviruses among each group’s total members.

We started analyzing the HERV and MaLR transcriptome in PBMCs from all samples, including those from pre-vaccinate individuals and those from individuals after the 2nd, 3rd, and 4th vaccine administration. Data showed that the public RNA-seq dataset reads mapped within the coordinates of 16,820 HERV hervgdb4 loci and 15,555 MaLR hervgdb4 loci (7.6% of the total loci) when considering both vaccinated and pre-vaccinated PBMCs samples. Additionally, reads mapped within the coordinates of 921 intact HERV proviruses from Vargiu et al. (29% of the total loci), which were analyzed in terms of distribution among the known phylogenetic groups (Figure 1). Elements from class II were very active, as we also previously observed [31]: among them, HERVK (HML-2) was the group with the highest percentage of expressed proviruses, with 47 out of 92 expressed loci. Within class I, HERVH, with a total of 278 active loci, was the group with the highest absolute number of expressed ReTe proviruses. Besides HERVH, HARLEQUIN, HERV9, HERVE, HERVIP, and HERVW were the most active among class I members, also in this case, showing a pattern of group expression similar to what we previously observed. Of note, 100% of loci from low-copy number groups HEPSI4, HUERPS1, LTR57, and PRIMLTR79 were expressed (these groups included only 1, 2, 1, and 1 loci, respectively).

Then, we focused on the loci expressed in pre-vaccinated samples, i.e., one day before the 1st vaccination, to study the basal HERV expression in PBMCs. In particular, we collected the transcript expression levels measured as Transcript per Million (TPM), finding 16 HERV proviruses highly expressed (average TPM ≥ 15), 16 mediumly expressed (average TPM < 15 and ≥9), 18 lowly expressed (average TPM < 9 and ≥5), and 117 very lowly expressed (average TPM < 5 and ≥1) (File S1). The analysis of the 16 highly expressed proviruses showed that the majority of them were intragenic integrations (Table 1).

**Figure 2:**
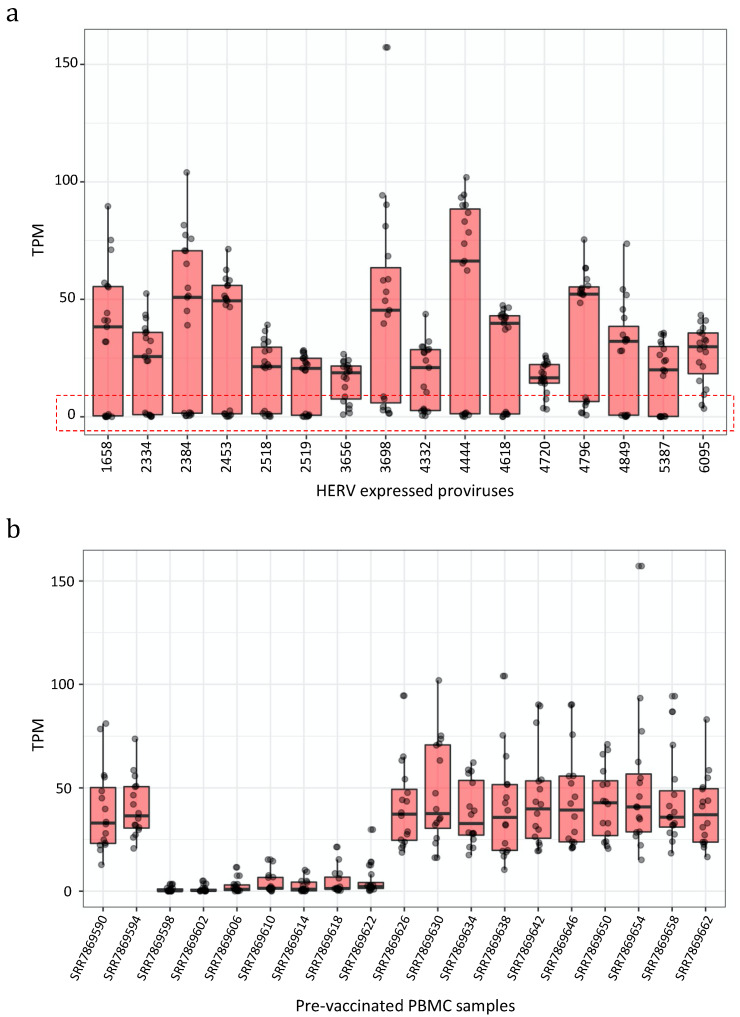
Expression values of the 16 most expressed proviruses. Boxplot of the expression values (measured as TPMs) of the 16 proviruses with the highest expression. (**a**) All proviruses showed a strong interpersonal variability being lowly or not expressed in some samples (red box). (**b**) We hence assessed the expression of the 16 proviruses among the individuals, finding 7 of them where the HERVs were not or only lowly expressed.

In fact, 11 proviruses were integrated within the intron of genes and 2 were integrated within a window of 10 kb from the nearest gene. Among those was also the HML-6 provirus at locus 19q13.43b (chr19:58305729–58315116), namely ERVK3-1 (ENSG00000142396), reported to be expressed in various healthy tissues and predicted to be protein-coding, within which we previously identified a Rec domain, suggesting the production of a Rec protein in PBMCs [44,45]. Next, we asked whether these 16 most expressed proviruses showed any interpersonal transcriptional variability, observing that it was very high. The considered HERV proviruses were expressed with elevated TPM values (highest TPM = 102) in some samples, but they were lowly or not expressed at all in some others (Figure 2a). We tried to understand how the TPM values of these lowly-expressed HERVs were distributed among the single pre-vaccinated samples, finding that in seven samples the expression of these 16 HERVs was overall indeed much lower than the other nine samples (Figure 2b).

To understand this fact better, we wanted to inquire whether this same interpersonal variability was present when considering the expression levels of other HERV and MaLR hervgdb4 fragments, as well as cellular genes, or if it was specific for these loci. For this reason, we analyzed the impact of the different expression patterns in these seven samples with Principal Component Analysis (PCA). The PCA confirmed, for both HERVs/MaLRs and genes, that the seven samples clustered differently from the other 12 samples The PC1 explained 83% and 75% of the variability among pre-vaccinated samples, based on HERV/MaLR and gene expression respectively (Figure S2). Due to the peculiar HERV/MaLR and gene expression in the mentioned seven samples, we could not properly explain this variability that could even be caused by technical bias. However, since the data were retrieved by publicly available transcriptome data that have been already obtained, checked for quality, edited, and published [42], we decided not to exclude these seven samples from the further analysis. Next, considering all 19 non-vaccinated samples, we observed that 167 HERV proviruses were expressed in PBMCs, of which 160 were expressed (with TPM ≥ 1) in at least 75% of individuals, five were expressed in at least 50% of individuals, and two were expressed in less than 50% of individuals. Of note, when considering the standard deviation of the TPM values among samples, we observed that they were higher especially for highly expressed proviruses (Figure S4). For example, the HERV with the highest interpersonal variability, in locus chr12:9832951–9837802 (ID 3698), had expression values from a minimum of 40 to a maximum of 157 TMP.

### 3.2. Analysis of Transcriptional Patterns Induced by Vaccine Administrations

To better understand if there is a correlation between HERV and MaLR transcriptional expression and vaccination against the Hantaan virus, we checked for the presence of a specific pattern of HERV/MaLR expression induced by the vaccine administration. Indeed, we previously reported HERV and MaLR transcriptional variation after the activation of the innate immunity [31], and we wanted to compare the observed modulation with a possible one specific for adaptive immune responses.

Keeping in mind that the vaccination may induce both innate and adaptive immunity, we wanted to understand the possible involvement of genes activated in the innate immune response. Since innate immunity has been linked to the expression of 44 genes known to give specific signatures of induced cytokine response [46], we firstly investigated their behavior in the samples obtained following the three vaccine administrations. The PCA (Figure S3) showed no sample clusters related to the expression of these genes after the vaccine administrations, confirming that they were not involved in patterns specific for adaptive immunity. This result suggested that eventual HERV/MaLR differential expression after vaccination is probably not linked to induced cytokine response.

**Figure 3:**
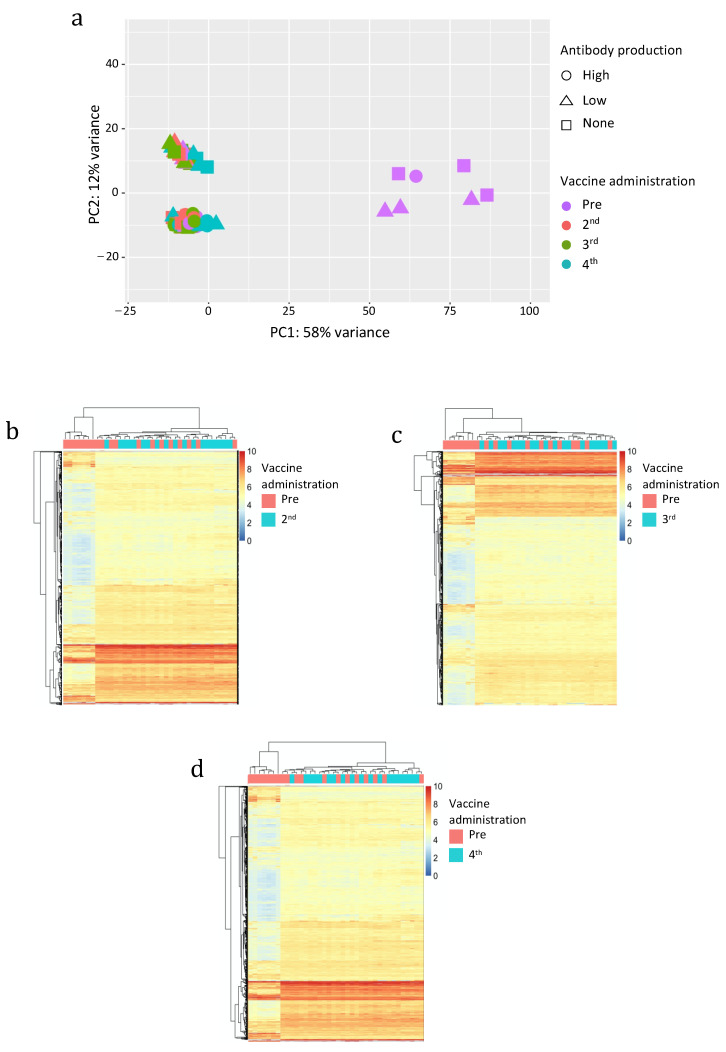
Principal Component Analysis (PCA) (**a**) and heatmaps of the overall similarity between pre-vaccinated and samples after the 2nd (**b**), 3rd (**c**), and 4th (**d**) vaccine administration. PCA was performed on rlog-normalized *hervgdb4* fragments expression data (**a**). The PC1 explains 58% of the overall variance. Heatmap. Hierarchical clustering was performed on the top 1500 *hervgdb4* fragments with the highest standard deviation (rows) based on rlog-normalized counts in each sample (columns). rlog-normalized counts are color-scaled from blue (minimum) to red (maximum). Correlation distance measure has been used in columns’ clustering. Samples are annotated by vaccine administration (red pre-vaccinated and light blue after the 2nd, 3rd, and 4th administration).

Then, we analyzed the variability among the samples attributable to the expression of hervgdb4 fragments by PCA (Figure 3a). The PC1 explained a high proportion of the variance across samples (58%), with seven samples clustering differently from all the others. Since these seven samples were pre-vaccinated ones, these clusters seemed to be somehow related to the vaccine administration. However, those seven samples were the same we already observed to have a low HERV/MaLR expression. Of note, no relationship between hervgdb4 expression and high- low- and non-responders was observed.

The other 69 samples were clustered into two groups across the PC2, but only 12% of the total variance explained this clustering. Such behavior was confirmed by the individual heatmap on pre-vaccinated samples and 2nd, 3rd and 4th vaccine administered samples (Figure 3b–d) since we did not observe any sample clustering related to the vaccine administration. In addition, we also performed hierarchical clustering of the samples excluding the seven low-HERV/MaLR expressing samples, and we still did not find specific signatures of HERV/MaLR expression induced by vaccines (data not shown).

### 3.3. Differential HERV and MaLR Expression After Vaccine Administration

**Figure 4:**
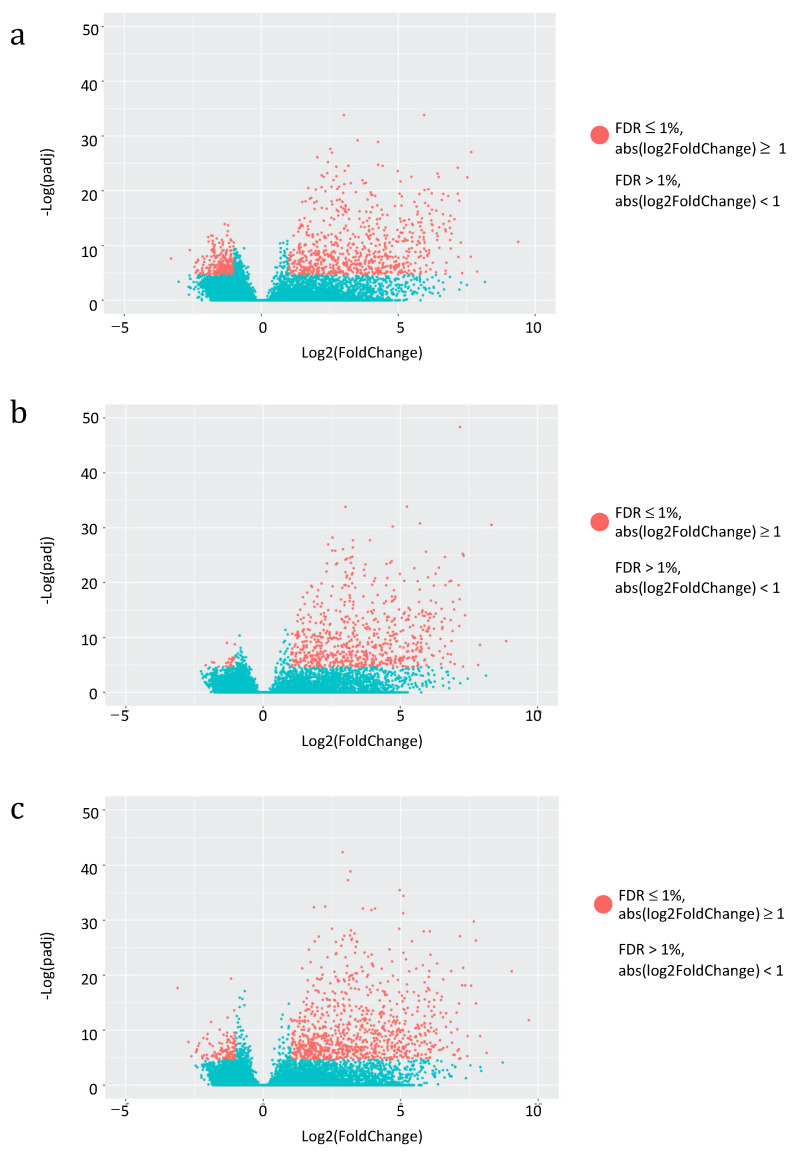
Differential HERV/MaLR expression after vaccine administrations. Volcano-plot of the differentially expressed hervgbd4 fragments (red dots) after the 2nd, 3rd and 4th vaccine administration are shown in (**a**), (**b**), and (**c**), respectively. Each dot represents individual hervgbd4 fragments, which spread according to the log2 fold change (*x*-axis), and the log10 adjusted *p*-values (*y*-axis).

Having assessed the global impact of vaccine administration on the overall HERV and MaLR transcriptome, we then asked for the specific effect on individual HERV and MaLR loci expression. We evaluated the hervgdb4 fragments for differential expression in three different combinations of conditions: (i) pre-vaccination and 2nd administration, (ii) pre-vaccination and 3rd administration, and (iii) pre-vaccination and 4th administration. We applied a statistical filter (FDR ≤ 0.01 and absolute values of log2 Fold Change ≥ 1) to identify the modulated hervgdb4 fragments, that we represented in a volcano plot. Particularly, hervgdb4 fragments that resulted as differentially expressed are indicated as red dots (Figure 4).

Interestingly, the great majority of hervgdb4 fragments were positively modulated, showing a general trend of HERV/MaLR up-regulation after each vaccine administration. Among the totality of expressed elements, 1032 hervgdb4 fragments (3.4%) were differentially expressed after the 2nd administration, 715 (1.3%) were differentially expressed after the 3rd administration, and 1012 (1.7%) were differentially expressed after the 4th administration (Table 2). When considering the hervgdb4 loci, we found that 609 loci were modulated after the 2nd, 396 after the 3rd, and 576 after the 4th administration. Finally, we found 23, 13, and 27 most intact ReTe proviruses differentially expressed after the 2nd, 3rd, and 4th administration, respectively (Table 2 and File S2).

**Figure 5:**
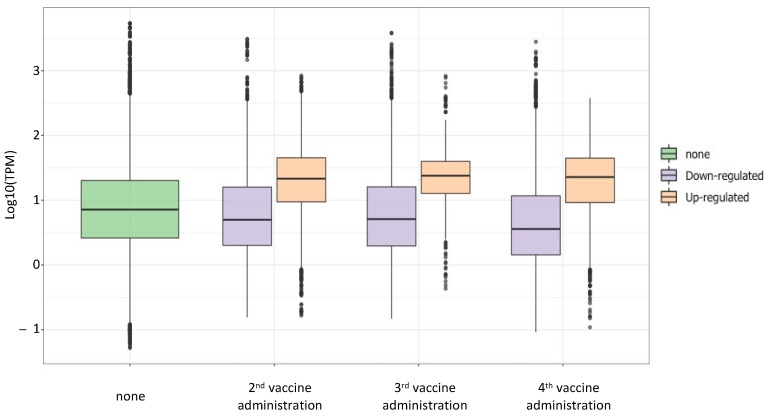
Expression levels of differentially expressed hervgdb4 after the 2nd, 3rd, and 4th vaccine administration. The boxplot represents the TMP values of the hervgdb4 fragments that were differentially expressed after each vaccine administration compared to the baseline (not vaccinated samples).

Since data showed an increase of the expression after the 2nd, 3rd, and 4th vaccination, we wanted to investigate if the HERV/MaLR expression levels gradually increased over time. We compared to the baseline level the HERV/MaLR TPM values before and after each vaccine administration (Figure 5) in a boxplot. The analysis demonstrated that the HERV/MaLR expression level after the 2nd, 3rd, and 4th administrations presented several HERV elements that were expressed to a higher level when compared to the pre-vaccination baseline. Differently, the analysis of the TPM values demonstrated that the overall expression level of such loci did not change when comparing their expression after the 2nd with their expression after the 3rd administration and the one after the 3rd with the one after the 4th administration.

**Figure 6:**
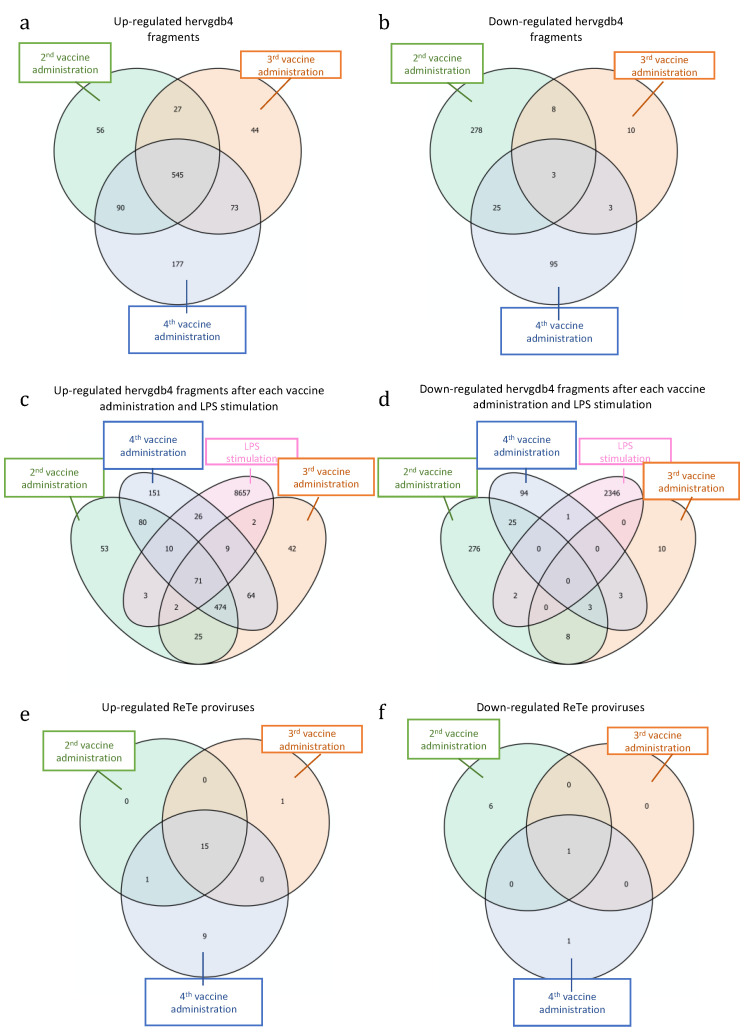
Differentially expressed HERVs and MaLR after each vaccine administration and LPS stimulation. Intersections of up-regulated (**a**) and down-regulated (**b**) *hervgdb4* fragments after each vaccine administration. Intersection of up-regulated (**c**) and down-regulated (**d**) *hervgdb4* fragments after each vaccine administrations and after LPS stimulation (data from Pisano et al. 2019 [32]). Up-regulated (**e**) and down-regulated (**f**) ReTe proviruses after all the vaccine administrations.

Next, we tried to understand if the differentially expressed elements may be specific signatures either for vaccination or for the various steps of the immune response to the vaccine. We searched for possible hervgdb4 fragments that were modulated after each single administration, as well as those that were modulated after all the administrations (Figure 6). The Venn diagram with the intersections of the up-regulated elements in the three considered conditions showed that a large fraction of them (545 hervgdb4 fragments) were differentially expressed after each vaccine administration (Figure 6a). Among these 545 hervgdb4 fragments, when considering the mean TPM values, we did not observe any individual increase or decrease in expression level after the 2nd, 3rd, and 4th administrations (data not shown). Moreover, we found 56, 44, and 177 hervgdb4 fragments that were only specifically up-regulated after the 2nd, 3rd, and 4th vaccination, respectively. Finally, 27 hervgdb4 fragments were up-regulated after both the 2nd and 3rd, 73 were up-regulated after both the 3rd and 4th vaccinations, and 90 were up-regulated after both the 2nd and 4th vaccinations (Figure 6a). However, when considering the mean TPM values, we did not observe any individual increase or decrease in expression level among the single hervgdb4 fragments up-regulated after the 2nd and 3rd, or after the 3rd and 4th, or after the 2nd and 4th vaccinations (data not shown).

When considering the down-regulated hervgdb4 fragments, only three of them were similarly modulated after each administration (Figure 6b). Additionally, among these three hervgdb4 fragments, when considering the mean TPM values, none of them showed an individual increase or decrease in expression levels at different time points (data not shown). We found 278, 10, and 95 hervgdb4 fragments that were only specifically down-regulated after the 2nd, then 3rd, and the 4th vaccination, respectively. Moreover, eight hervgdb4 fragments were commonly down-regulated after the 2nd and 3rd, three were commonly down-regulated after the 3rd and 4th, and 25 were commonly down-regulated after the 2nd and 4th vaccinations (Figure 6b). When considering the mean TPM values, we did not observe any individual increase or decrease in expression level in the hervgdb4 fragments down-regulated after the 2nd and 3rd, or after the 3rd and 4th, or after the 2nd and 4th vaccinations (data not shown). Then, we checked for intersection with the previously identified hervgdb4 fragments modulated after LPS stimulation [31]. In this case, we found that 71 hervgdb4 fragments were up-regulated after the vaccination and after LPS stimulation (Figure 6c), while none of the hervgdb4 fragments were down-regulated after both vaccine and LPS injection (Figure 6d). This highlights how the HERV and MaLR modulation is different between the two conditions.

Furthermore, we analyzed the ReTe proviruses that were up-regulated or down-regulated after each vaccine administration. Among the up-regulated ReTe proviruses (Figure 6e–f), 15 were up-regulated after each vaccination, one after the 3rd, and nine after the 4th administration. Only one provirus was instead down-regulated after each administration. Hence, we further analyzed the 16 ReTe proviruses that were up-regulated or down-regulated after all vaccine administrations (Table 3). Among them, 12 were intragenic integrations, including five proviruses that were intronic integrations and one provirus that was an exonic integration. Importantly, the majority of the genes nearby the proviral integration were not differentially expressed, with the only exception of the gene RPS23, up-regulated after the vaccine administration.

Finally, to better understand the correlation between HERV/MaLR and gene modulation in immunity, we searched for possible patterns of gene expression similar to the one of HERV/MaLR-gene differential expression. We firstly performed Gene Set Enrichment Analysis (GSEA), an algorithm that identifies statistically enriched gene sets in transcriptomic data, to quantify the enrichment of genes belonging to known cell immune-related pathways after the vaccine administrations [41,47]. The enrichments were measured as Normalized Enrichment Scores (NES). Comparing pre-vaccinated sample with the ones following the 2nd and 3rd vaccine administration, we observed an enrichment of some immune-related pathways (Figure S5), but these pathways were mostly diverse among the two time points, while we did not find major pathway immune activation following the 4th administration (Figure S4).

Secondly, we searched for possible correlations between these enriched pathways and the differentially expressed hervgdb4 loci. Results showed that 354 out of the 1032 hervgdb4 loci differentially expressed after the 2nd vaccine administration correlated to two genes included in pathways enriched after that administration. These genes were *RPL6* and *RPS2*, both associated with the MYC targets pathway. Moreover, 7 out of 715 hervgdb4 loci differentially expressed after the 3rd vaccine administration correlated to the CA7 gene, involved in pathways related to UV response and Complement, both enriched after 3rd vaccine administration. Finally, 113 out of 1012 hervgdb4 loci differentially expressed after the 4th vaccine administration correlated to six genes (*UQCRH*, *ATP5F1D*, *COX7C*, *COX6B1*, *UQCRQ*, and *LDHB*) belonging to the oxidative phosphorylation pathway, which was enriched after that administration. None of the hervgdb4 loci correlated to that genes after each vaccine administrations were included in the ones found to be modulated after all the other administrations.

## 4. Discussion

We used an RNA-seq approach to obtain an overview of the specific HERV and MaLR transcriptome in PBMCs before and after the activation of the adaptive immune response, as triggered by the administration of the virus-inactivated vaccine. Moreover, we were willing to search for specific patterns of HERV and MaLR expression linked to the various stages of immunity, where we compared our results with the HERV and MaLR modulation observed in innate immunity settings from our previous work [31]. The present work was based on transcriptome data from PBMC samples, and we found a basal HERV/MaLR expression in these cells. Indeed, about 7% of hervgdb4 fragments and 8% of hervgdb4 loci were expressed in PBMCs, similar to what we observed in our previous work [31]. Several groups from all three retroviral classes were transcriptionally active. Among the groups belonging to class II, the HML-2 group was the most transcriptionally active, but despite that, the HML-2 proviruses were not the most expressed when considering TPM expression values. This indicates that a large number of HML-2 are transcribed, but with medium or low expression levels, and none of them were highly expressed. Among class II elements, we found expressed the HML-6 locus at coordinates chr19:58305729–58315116, known to include a Rec domain within its sequence. The Rec proteins have often been investigated for a suggested contribution to cancer [13,23,48]. As the transcript of this HML-6 locus has been predicted to be protein-coding [44], further studies may help to understand the possible impact of such protein in human physiopathology. Noteworthy, the majority of highly expressed proviruses were intragenic integrations, and it could be interesting to investigate a possible mutual HERV/co-localized genes influence their transcriptional activity.

When considering the pre-vaccinated individuals, we observed an important interpersonal variability. Indeed, the expression of HERV/MaLR and cellular genes in seven individuals was lower than that in the other 12. This particular HERV/MaLR expression pattern explained about 83% of the variance among samples, according to the PCA, even if further information is needed to explain such variation. Particularly, the fact that we did not observe a similar interpersonal variability in PBMCs in our previous work [31] does not allow us to exclude a technical bias in collecting and processing the samples. Nonetheless, up to now, this is the first study reporting a similar interpersonal variability in gene or HERV/MaLR expression in PBMCs.

Several studies have observed specific HERV/MaLR individual loci up-or down-regulation after viral infection [45], acute inflammation [29,30], cancer [15,49,50], or other diseases [51,52]. We tried to understand if there was a specific pattern of HERV/MaLR activation at different immunity stages. Different from what we observed in vivo after LPS stimulation, we did not find clear patterns of HERV/MaLR activation as induced by the vaccine. However, we found several HERVs and MaLRs differentially expressed after the three vaccine administrations (2nd, 3rd, and 4th), out of four. The majority of differentially expressed HERVs and MaLRs were up-regulated, and their expression increased in samples after vaccination, with respect to the not-vaccinated ones. Despite that, we did not observe a gradual increase of TMP values over time, but the HERV/MaLR expression was stable among all the administrations. The number of differentially expressed HERV and MaLR after vaccination was reduced in comparison to what we observed after LPS stimulation [31], but our data clearly showed that different loci were differentially expressed after the 2nd, 3rd, and 4th vaccine administration.

In the comparison between HERV/MaLR modulation after LPS stimulation and vaccines, it is possible that different time points in collecting the samples may be causative of a different HERV/MaLR activation. Indeed, in the LPS study, samples were collected 2 h from the injection, while in this study samples were collected 72 h after the vaccinations. Hence, the effect on HERV/MaLR activation could be lower 72 h after treatment, and/or the vaccine may cause a lower activation of the innate immune response. The HERV and MaLR up-regulation in innate immunity seemed to be linked to genes induced by cytokines and, in that case, we also observed a strong activation of the 44 immune-related genes [46]. Instead, after vaccine administration, this immune-associated induction lacks, and we did not find any activation of the 44 immune-related genes. Nevertheless, the activation of innate immune response cannot be excluded, as the Hantavax™ vaccine is formulated with alumn adjuvant [32,53], which may boost it. Moreover, it is important to also keep in mind that the response to LPS (Gram-bacteria) and to an inactivate vaccine may likely be different in stimulating innate immunity. Hence, even if the up-regulation of specific HERV/MaLR is not sufficient to discriminate settings of activation of the adaptive immune response triggered by the inactivated vaccine, the up-regulation of some HERV and MaLR loci may be indicative of specific immunity settings, for example related to Gram– infection, or subsequential inactivated vaccination.

Finally, as we previously found HERV and MaLR loci modulated in innate immunity correlated to immune-related genes [31], we searched for differentially expressed HERVs and MaLRs correlated to differentially expressed genes involved in immune pathways of the adaptive response. However, none of the hervgdb4 loci correlated to these genes after all the vaccine administrations were common to the ones differentially expressed after innate immunity stimulation or included in the ones found to be modulated after all the vaccine administrations (data not shown).

Further studies are needed to better understand the HERV and MaLR expression during host immune responses. As the HERV and MaLR expression is correlated to specific settings in inflammatory contexts, studies including large cohorts of patients may allow estimating whether and how much HERVs and MaLRs could serve as biomarkers for the different immune phases in acute and chronic infections. Moreover, a complete characterization of the HERV and MaLR basal expression, including interpersonal differences, and their dynamic activation in different stages of immunity, would be an important starting point to explore the possible usage of HERVs and MaLR as a potential therapeutic target.

## 5. Conclusions

In conclusion, our data showed a consistent HERV and MaLR basal expression in PBMCs, characterized by a certain interpersonal variability. We identified individual HERV and MaLR loci differentially expressed after the 2nd, 3rd, and 4th administrations of an inactivated vaccine against the Hantaan virus. The majority of differentially expressed HERVs and MaLRs were up-regulated, and the expression of such up-regulated elements increased in samples after vaccination, with respect to the not-vaccinated ones. Our data showed that the expression of 545 hervgdb4 fragments increased after the vaccination, while the expression of three hervgdb4 fragments decreased after the vaccination. Furthermore, the differential expression of several individual HERV and MaLR loci was specific to each vaccine administration and correlated to different genes and immune-related pathways. The identification of specific HERV and MaLR activation in various stages of immunity may help further studies focused on a possible link between HERV/MaLR expression and acute and chronic diseases and infections.

## Figures and Tables

**Table 1:** 16 highly expressed HERV proviruses in PBMCs and their context of insertion.

ReTe ID	Locus	Group	TPM	Genomic Context	Cellular Neighbor Gene	Relative Strand
4444	chr17:35500762–35508355	HERVE	52.22	Intronic	*SLFN12L*	−
3698	chr12:9832951–9837802	HERVH	45.69	Intronic	*KLRF1*	−
2384	chr6:158611703–158621178	HERVFA	41.77	Intronic	*TMEM181*	−
4796	chr19:58305729–58315116	HML-6	37.60	Downstream	*ZNF8*	+
2453	chr7:30572445–30579657	HERV4	35.01	Intronic	*GARS1*	+
1658	chr4:153690317–153693920	HML-3	33.37	Intronic	*TLR2*	−
4618	chr19:11942587–11948985	HERVE	27.21	Intronic	*ZNF700*	−
6095	chr1:169683482–169691301	HERVH	26.64	Intronic	*SELL*	−
4849	chr20:49281128–49287343	HERVIP	25.92	Intergenic	*NA*	
2334	chr6:130192281–130199095	HERVH	22.05	Intronic	*SAMD3*	−
2518	chr7:64679995–64686561	HERVH	18.09	Intronic/Exonic	*ZNF107*	+
4332	chr16:3079068–3081318	HML-3	17.35	Intergenic	*NA*	
5387	chrX:53160069–53162218	HML-6	17.33	Intergenic	*NA*	
4720	chr19:38823108–38837433	HERV9	16.67	Intronic	*ECH1*	−
2519	chr7:64834895–64840158	HERVFC	15.26	Downstream	*ZF138*	−
3656	chr11:121632566–121643491	HERVH	15.40	Downstream	*SORL1*	−

**Table 2:** Overview of MaLR/HERVs modulation after vaccine administrations.

Vaccine Administration	Database	Up-Regulated	Down-Regulated
2nd	hervgdb4 fragments	718	314
	hervgdb4 loci	657	275
	ReTe proviruses	16	7
3rd	hervgdb4 fragments	691	24
	hervgdb4 loci	635	24
	ReTe proviruses	16	1
4th	hervgdb4 fragments	885	127
	hervgdb4 loci	808	117
	ReTe proviruses	25	2

**Table 3:** Description of the 16 ReTe proviruses differentially expressed after all vaccine administrations

Chr	Start	End	Strand	Length	ReTe ID	Group	Neighbor Gene
chr1	155650288	155659631	−	9343	6072	HERV4	*YY1AP1* ^1^
chr10	18570092	18577466	+	7374	3200	HERVIP	*CACNB2* ^1^
chr11	58769831	58777331	+	7500	3503	HERV1	-
chr17	11971744	11978102	+	6358	4426	HERVH	*ZNF18* ^2^
chr19	36149712	36161023	−	11311	4713	HERVH	*CAPNS1* ^2^
chr2	69789472	69799355	−	9883	565	HUERSP3	*ANXA4* ^3^
chr22	16611312	16616782	+	5470	6262	HERVH	-
chr3	107564215	107572787	−	8572	1058	HERVH	*BBX* ^3^
chr3	193599956	193613333	−	13377	1278	HEPSI1	*OPA1* ^3^
chr4	25238665	25247155	−	8490	1350	HERV9	*PI4K2B* ^3^
chr4	53236811	53255667	−	18856	1405	HML2	*SCFD2* ^3^
chr4	139442392	139449817	+	7425	1638	HERVL	*RAB33B* ^2^
chr5	70512460	70531584	+	19124	1874	THE	-
chr5	82267546	82273706	−	6160	1892	HARLEQUIN	*RPS23* *^,4^
chr6	148639772	148645510	+	5738	2371	HERVH	-
chr7	43853008	43866752	−	13744	2476	HML3	*MRPS24* ^1^

^1^ HERV down-stream the gene, ^2^ HERV up-stream the gene, ^3^ HERV within the intron of the gene, ^4^ HERV within the exon of the gene, * Up-regulated.

## Data Availability

The RNA-seq dataset is publicly available in the Gene Expression Omnibus (GEO) repository with the accession ID GSE120115.

## Supplementary Materials

The following are available online at https://www.mdpi.com/article/10.3390/biology10050405/s1, Figure S1: Experimental design of Differential Expression analysis, Figure S2: Differences in HERV/MaLR and gene expression among pre-vaccinated individuals, Figure S3: Principal Component Analysis (PCA) of all samples according to the expression of 44 genes, Figure S4: Expression values of the HERV proviruses with the highest standard deviation, Figure S5: Cellular pathways enriched after the 2nd, 3rd and 4th vaccine administrations, File S1: Expression levels of expressed ReTe proviruses, File S2: Differentially expressed HERV/MaLR.
