# Supplementary material for: Human Endogenous Retroviruses (HERVs) and Mammalian Apparent LTRs Retrotransposons (MaLRs) Are Dynamically Modulated in Different Stages of Immunity
Source: Biology (Basel). 2021 May 5;10(5):405. doi: 10.3390/biology10050405 (PMC8147956; doi:10.3390/biology10050405)
Supplement: Supplementary file 1 [file biology-10-00405-s001.zip › Supplementary_figures.pdf]

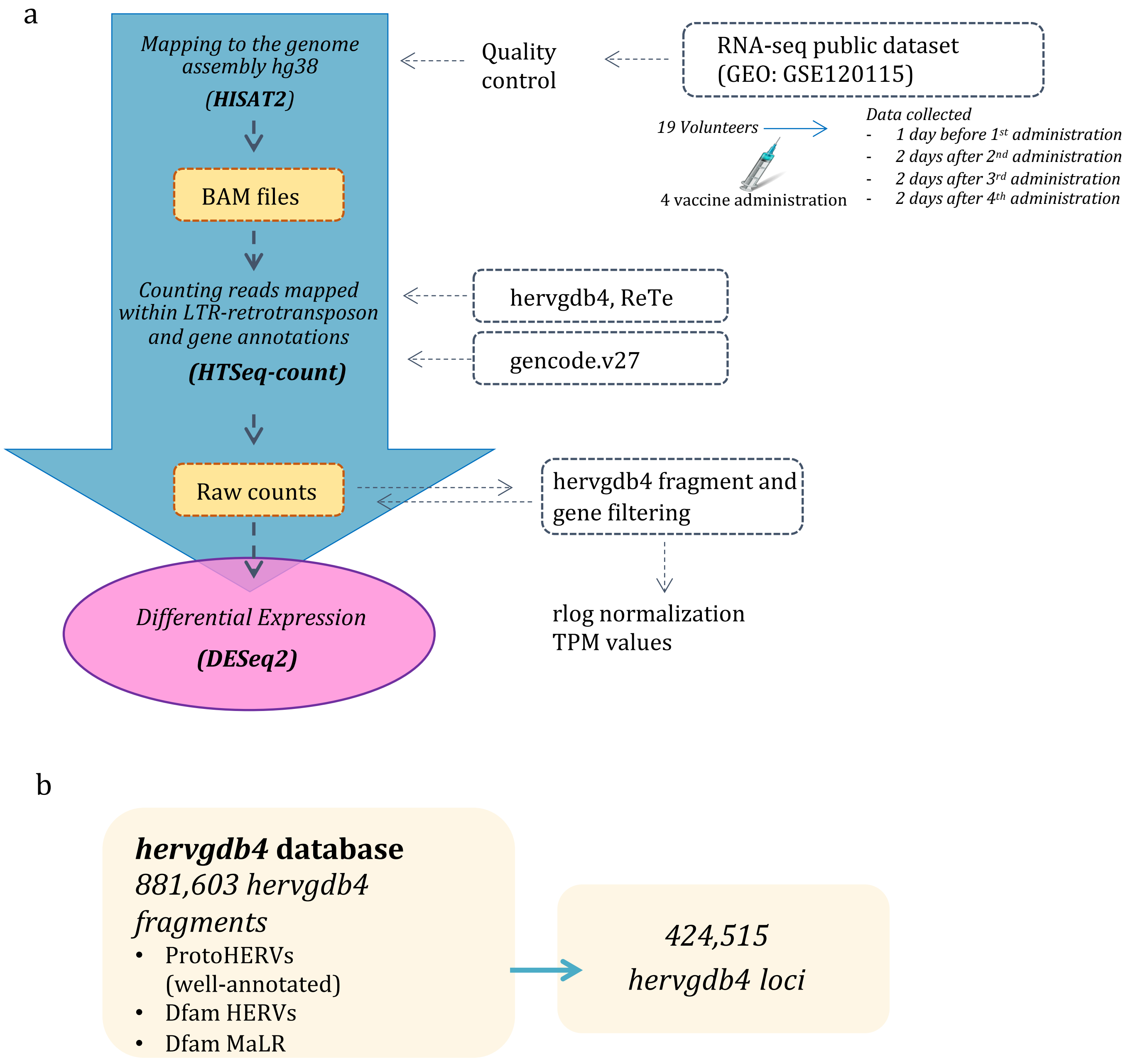

Expressed elements	hervgdb4 fragments	hervgdb4 loci	HERV proviruses
HERV	29535	16820	921
MaLR	26732	15555	NA
tot	56267	32375	921

Figure S1. Experimental design of Differential Expression analysis. A RNA-seq workflow has been applied to the identification of modulated HERVs and MaLRs: the input files used are listed in blue boxes (a); while the composition of hervgdb4 database is schematized below (b). The amount of expressed hervgdb4 fragments and loci that have been obtained by filtering the raw counts and are summarized in the table.

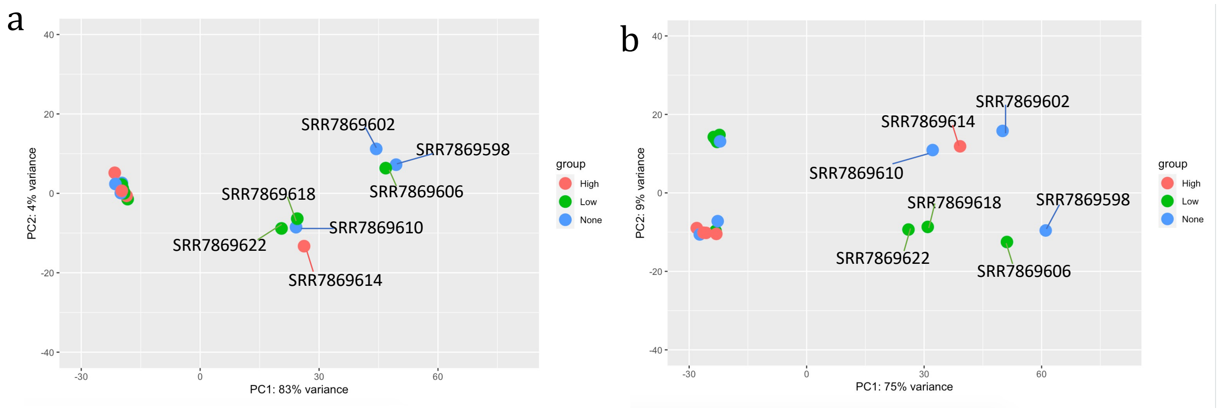

Figure S2. Differences in HERV/MaLR and gene expression among pre-vaccinated individuals. The Principal Component Analysis (PCA) of samples showed the presence of specific pattern of HERV/MaLR (a) and human gene (b) expression. In both cases, 7 of the 19 samples clustered differently accordingly to the PC1, which explained the 83% and 75% of the variability among pre-vaccinated samples, respectively.

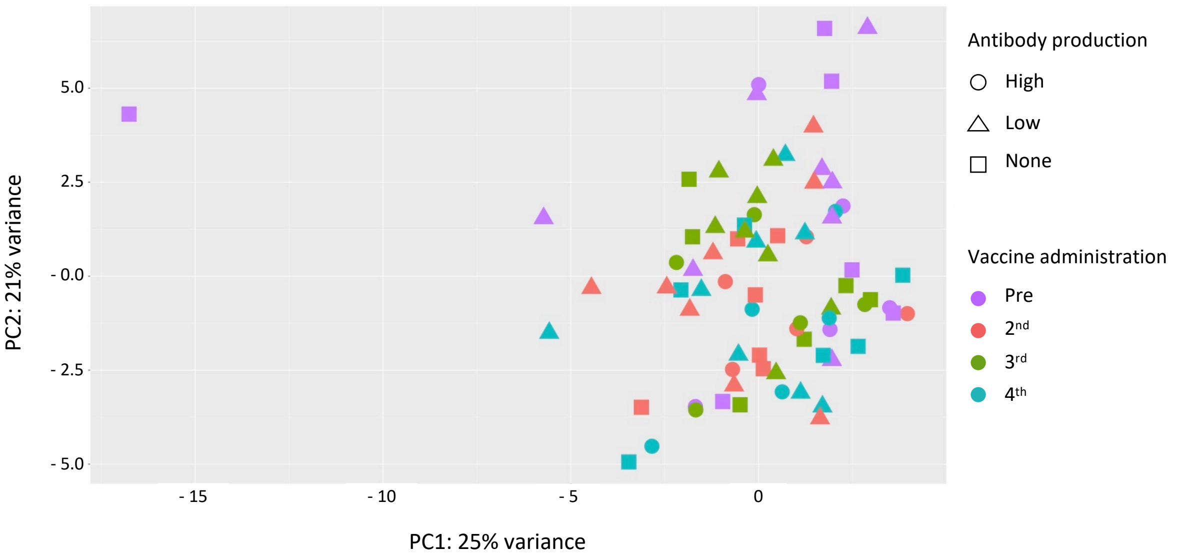

Figure S3. Principal Component Analysis (PCA) of all samples according to the expression of 44 genes known to be involved in innate immunity. PCA was performed on rlog-normalized expression data, and did not show any evident division between vaccinated and not-vaccinated samples, or between samples showing different antibody production.

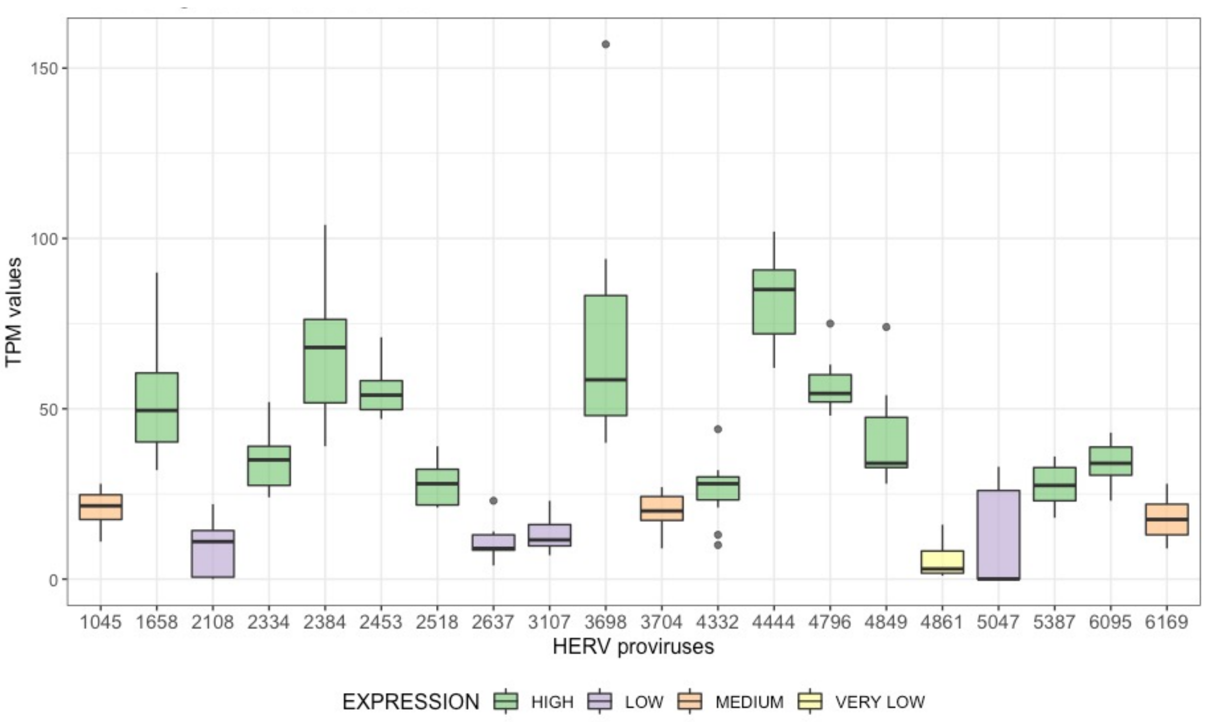

Figure S4. Expression values of the HERV proviruses with the highest standard deviation. The boxplots of Transcripts per Million (TPM) expression values of the 20 HERV proviruses with the highest standard deviation among samples showed that the HERV with higher interpersonal variability are also those showing the highest expression.

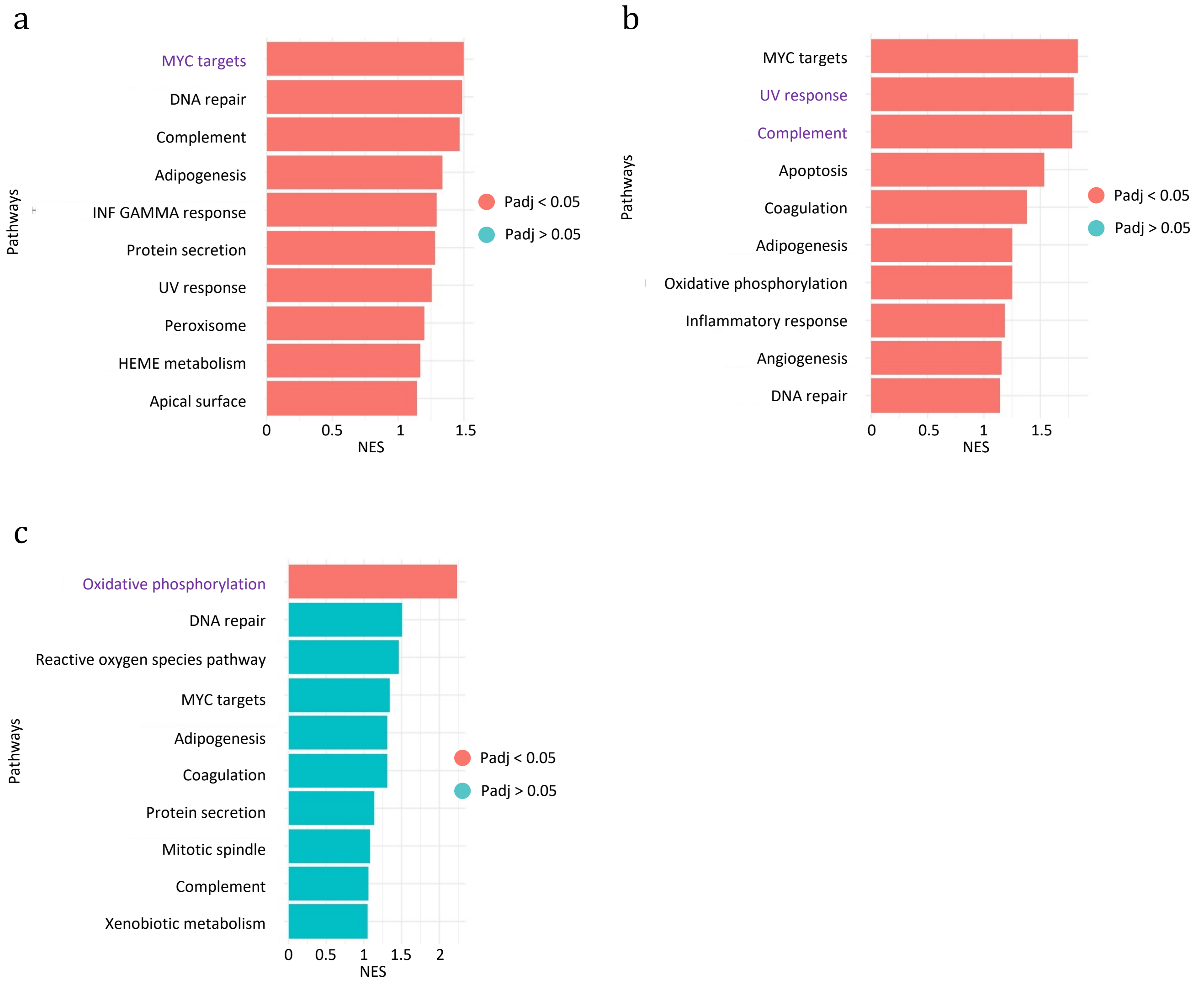

Figure S5. Cellular pathways enriched after the 2nd (a), 3rd (b) and 4th (c) vaccine administrations. The violet-marked pathways are those with genes correlated to differentially expressed HERVs and MaLRs.
